# CD4 ^+^ T Cell‐Intrinsic Arg1 Regulates Th1 Dynamics in Influenza: Functional and Translational Implications for Infection and Cancer Inflammation

**DOI:** 10.1111/all.70249

**Published:** 2026-02-05

**Authors:** Anishaa Balaji, Sophia N. Karagiannis, Alexandra J. McCraw

**Affiliations:** ^1^ St. John's Institute of Dermatology, School of Basic & Medical Biosciences, & KHP Centre for Translational Medicine, Guy's Hospital King's College London London UK; ^2^ Breast Cancer Now Research Unit, School of Cancer & Pharmaceutical Sciences King's College London, Guy's Cancer Centre London UK

**Keywords:** basic immunology, infections, T cells

Timely induction and subsequent contraction of interferon‐gamma (IFN‐γ)‐producing CD4^+^ T cells is critical for infection clearance and re‐establishing homeostasis, respectively [1]. Insufficient downregulation of IFN‐γ signaling and delayed induction of interleukin‐10 (IL‐10) by Th1 cells can lead to persistent normal tissue injury, as observed in cases of severe COVID‐19 [2]. Understanding the factors that regulate these processes and their temporal dynamics is important for effective disease control.

Amino acid metabolism, a well‐documented regulator of immune responses in cancer, autoimmunity and inflammatory conditions [3], is associated with Th1 immune responses. Amino acid–dependent pathways, including L‐arginine metabolism, shape T‐cell differentiation and promote Th1 responses. Moreover, coordinated crosstalk between L‐arginine metabolism, vitamin D signaling, and cholesterol biosynthesis can drive complement activation and induce IFN‐γ–producing CD4^+^ T cells during infection [2, 4, 5].

Arginase 1 (Arg1), the cytosolic isoform of the Arginase enzyme that converts L‐arginine into ornithine and urea, can be induced by Th2 cytokines in alternatively‐activated macrophages and linked to tumor‐associated immunosuppression [6]. However, its expression in T cells and in T cell responses to infection has been controversial.

West et al. [7] identified Arg1 as one of the most highly‐induced genes following influenza infection. T cell‐specific conditional knockout (CKO) of Arg1 in a murine model of influenza demonstrated expedited induction of actively proliferating CD4^+^ T cells in the lungs and subsequent resolution of virus‐specific Th1 responses compared to WT mice (Figure 1) [7]. The role of Arg1 in regulating CD4^+^ T cell response kinetics appeared independent of Arg1 and its mitochondrial isoform Arg2 in CD8^+^ T cells [7]. Arg1 CKO in CD4^+^ T cells also resulted in reduced glutaminolysis leading to altered IFN‐γ and IL‐10 production, an effect which was not observed following Arg1 CKO in CD8^+^ T cells. Arg1 expression therefore limits initial CD4^+^ T cell clonal proliferation following viral infection and delays transition to the IL‐10–producing contraction phase in mice and humans. Arg1 is thus shown as a key metabolic checkpoint and therapeutic target in T cells that fine‐tunes immune responses and limits tissue pathology in viral infections. Arg1‐dependent regulation to CD4^+^ T cells, without Arg2 involvement and with different outcomes in CD8^+^ T cells, may stem from distinct metabolic demands or compartmentalised arginine use in the cytosol versus mitochondria. Such divergence suggests lineage‐specific arginine flux, with CD4^+^ T cells relying on cytosolic Arg1, whereas CD8^+^ T cells use alternative mitochondrial pathways that circumvent Arg1 restriction.

**FIGURE 1:**
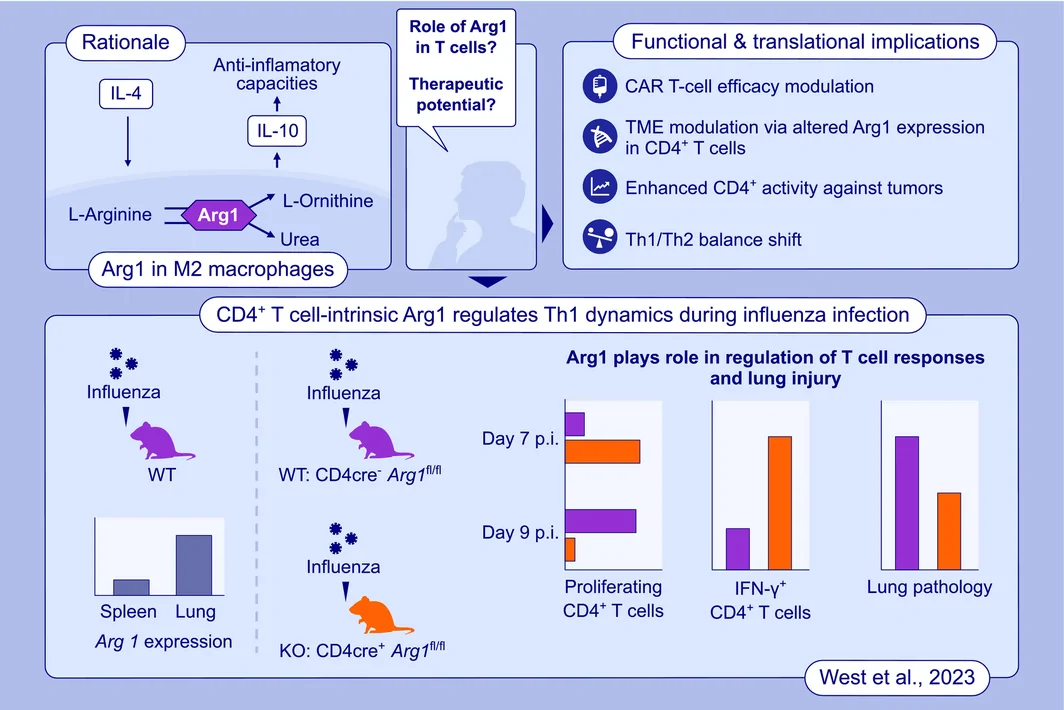
Arg1 plays a key role in regulating T cell induction and timely cessation in infections as exemplified by the enhanced CD4^+^ T cell proliferation, viral clearance, and earlier contraction of IFN‐γ producing CD4^+^ T cells in Arg1 KO mouse influenza models. Moreover, the role of Arg1 in modulating the Th1 response kinetics may have important implications in elucidating immunosuppressive tumor‐associated T cell characteristics, allergic sensitization, and additional targeted therapeutic applications. Arg1, Arginase 1; IFN‐γ, Interferon gamma; IL‐4, interleukin 4; IL‐10, interleukin 10; KO, Knock Out; TME, tumour microenvironment; WT, wild type.

Exploring the molecular underpinnings of metabolic regulation of Th1 responses to infections could yield insights into immune dysregulation in cancer, allergy and inflammatory conditions (Figure 1). Altered glutamine metabolism is a hallmark of cancer, and glutamine blockade is linked to improved anti‐tumor immunity [8]. Arg1 in tumors is expressed by immunomodulatory myeloid cells [6]. It shapes nutrient flux through regulation of L‐arginine metabolism, indirectly affecting polyamine synthesis and T cell fitness under nutrient‐deprived conditions which are typical of tumors [6]. Within the immunosuppressive tumour microenvironment, Arg1 expression in CD4^+^ T cells may limit clonal expansion and prolonged inflammatory cytokine production, restricting anti‐tumor immunity. Furthermore, Arg1 may influence the balance between pro‐inflammatory and regulatory CD4^+^ T cell states, impacting chronic inflammation and tumour tolerance. Clarifying how Arg1 is regulated in human CD4^+^ T cells across cancer, infection, and allergic disease is essential for assessing its therapeutic potential. Human and mouse T cells may use distinct regulatory and metabolic pathways, altering Arg1 induction and its impact on effector function or Th1/Th2 balance. Species differences underscore this need; for example, murine red cell Arg1 activity is far lower than in humans [9], highlighting the importance of validating Arg1‐targeted strategies directly in human immune cells.

If Arg1 expression and functions in cancer‐associated T cells are explored and elucidated, Arg1‐directed treatments could potentially enhance CD4^+^ T cell effector function while diminishing Arg1‐expressing myeloid cell‐associated immunosuppression. This may open translational opportunities including targeted CAR T‐cell therapies exploiting Arg1‐specific immune responses in prolonged inflammation following infections. Given the diverse and cell type‐dependent roles of Arg1, implementing cell type‐specific interventions [5, 10]. Furthermore, if induction of Arg1 by Th2 cytokines in CD4^+^ T cells can be confirmed, it would be valuable to elucidate whether this enzyme also regulates Th1 dynamics. Modulating this pathway could influence the Th1/Th2 balance, with potential implications for allergic sensitization and therapy across different immunological diseases.

## Funding

The authors have nothing to report.

## Conflicts of Interest

Anishaa Balaji reports grants from Apollo Therapeutics. Sophia N. Karagiannis reports grants from Breast Cancer Now, Worldwide Cancer Research, and King's Health Partners Centre for Translational Medicine during the conduct of the study as well as grants and other support from Epsilogen Ltd. and grants from Apollo Therapeutics, Cancer Research UK, the Wellbeing of Women, The Medical Research Council, The Biotechnology and Biological Sciences Research Council, Cancer Research UK City of London Centre, British Skin Foundation, and the National Institute for Health Research outside the submitted work, is academic founder and shareholder of Epsilogen Ltd. and has authored patents on antibody technologies for cancer. Alexandra J. McCraw reports grants from Worldwide Cancer Research during the conduct of the study.

## Data Availability

Data sharing not applicable to this article as no datasets were generated or analysed during the current study.

## References

[1] R. T. Gazzinelli , M. Wysocka , S. Hieny , et al., “In the Absence of Endogenous IL‐10, Mice Acutely Infected With Toxoplasma Gondii Succumb to a Lethal Immune Response Dependent on CD4+ T Cells and Accompanied by Overproduction of IL‐12, IFN‐Gamma and TNF‐Alpha,” Journal of Immunology 157, no. 2 (1996): 798–805. 8752931

[2] D. Chauss , T. Freiwald , R. McGregor , et al., “Autocrine Vitamin D Signaling Switches Off Pro‐Inflammatory Programs of T(H)1 Cells,” Nature Immunology 23, no. 1 (2022): 62–74. 10.1038/s41590-021-01080-3 PMC7612139 34764490

[3] Z. Lin , C. Chang , S. Zhao , L. Fang , and P. Wang , “Amino Acids Shape the Metabolic and Immunologic Landscape in the Tumor Immune Microenvironment: From Molecular Mechanisms to Therapeutic Strategies,” Cancer Biology & Medicine 22, no. 7 (2025): 726–746. 10.20892/j.issn.2095-3941.2025.0115 PMC12302275 40708274

[4] E. Perucha , R. Melchiotti , J. A. Bibby , et al., “The Cholesterol Biosynthesis Pathway Regulates IL‐10 Expression in Human Th1 Cells,” Nature Communications 10, no. 1 (2019): 498. 10.1038/s41467-019-08332-9 PMC6353904 30700717

[5] R. Geiger , J. C. Rieckmann , T. Wolf , et al., “L‐Arginine Modulates T Cell Metabolism and Enhances Survival and Anti‐Tumor Activity,” Cell 167, no. 3 (2016): 829–842. 10.1016/j.cell.2016.09.031 PMC5075284 27745970

[6] V. Bronte , P. Serafini , C. De Santo , et al., “IL‐4‐Induced Arginase 1 Suppresses Alloreactive T Cells in Tumor‐Bearing Mice,” Journal of Immunology 170, no. 1 (2003): 270–278. 10.4049/jimmunol.170.1.270 12496409

[7] E. E. West , N. S. Merle , M. M. Kamiński , et al., “Loss of CD4(+) T Cell‐Intrinsic Arginase 1 Accelerates Th1 Response Kinetics and Reduces Lung Pathology During Influenza Infection,” Immunity 56, no. 9 (2023): 2036–2053. 10.1016/j.immuni.2023.07.014 PMC10576612 37572656

[8] R. D. Leone , L. Zhao , J. M. Englert , et al., “Glutamine Blockade Induces Divergent Metabolic Programs to Overcome Tumor Immune Evasion,” Science 366, no. 6468 (2019): 1013–1021. 10.1126/science.aav2588 PMC7023461 31699883

[9] S. K. Heuser , J. Li , Z. Li , et al., “Divergent Roles of Red Cell Arginase in Humans and Mice: RBC Arg1 KO Mice Show Preserved Systemic l‐Arginine Bioavailability and Infarct Size In Vivo,” Redox Biology 86 (2025): 103768. 10.1016/j.redox.2025.103768 PMC12328693 40729962

[10] Z. Yang and X. F. Ming , “Functions of Arginase Isoforms in Macrophage Inflammatory Responses: Impact on Cardiovascular Diseases and Metabolic Disorders,” Frontiers in Immunology 5 (2014): 533. 10.3389/fimmu.2014.00533 PMC4209887 25386179

